# ACTH-like Peptides Compensate Rat Brain Gene Expression Profile Disrupted by Ischemia a Day After Experimental Stroke

**DOI:** 10.3390/biomedicines12122830

**Published:** 2024-12-13

**Authors:** Ivan B. Filippenkov, Yana Yu. Shpetko, Vasily V. Stavchansky, Alina E. Denisova, Leonid V. Gubsky, Lyudmila A. Andreeva, Nikolay F. Myasoedov, Svetlana A. Limborska, Lyudmila V. Dergunova

**Affiliations:** 1 Laboratory of Human Molecular Genetics, National Research Center “Kurchatov Institute”, Kurchatov Sq. 2, 123182 Moscow, Russia; yana.sch2014@yandex.ru (Y.Y.S.); bacbac@yandex.ru (V.V.S.); andr-la.img@yandex.ru (L.A.A.); myasoedov-nf.img@yandex.ru (N.F.M.); limbor.img@yandex.ru (S.A.L.); dergunova-lv.img@yandex.ru (L.V.D.); 2 Department of Biotechnology, Mendeleev University of Chemical Technology of Russia, Miusskaya Sq., 9, Building 1, 125047 Moscow, Russia; 3 Department of Neurology, Neurosurgery and Medical Genetics, Pirogov Russian National Research Medical University, Ostrovitianov Str. 1, 117997 Moscow, Russia; dalina543@gmail.com (A.E.D.); gubskii@mail.ru (L.V.G.); 4 Federal Center for the Brain and Neurotechnologies, Federal Biomedical Agency, Ostrovitianov Str. 1, Building 10, 117997 Moscow, Russia

**Keywords:** ischemic stroke, ACTH(6–9)PGP, ACTH(4–7)PGP (Semax), RNA-Seq, differential expressed genes, gene network

## Abstract

**Background:** Ischemic stroke results from a disruption of cerebral blood flow. Adrenocorticotropic hormone (ACTH) serves as the basis for the creation of synthetic peptides as neuroprotective agents for stroke therapy. Previously, using RNA-Seq we first revealed differential expressed genes (DEGs) associated with ACTH(4–7)PGP (Semax) and ACTH(6–9)PGP peptides under cerebral ischemia conditions. Analysis was carried out at 4.5 h after transient middle cerebral artery occlusion (tMCAO) model in the ipsilateral frontal cortex of a rat brain. **Methods:** Here, we analyzed the penumbra-associated frontal cortex of rats and actions under the same peptides at 24 h after tMCAO using RNA-Seq. **Results:** 3774 DEGs (fold change > 1.5 and *Padj* < 0.05) were identified under ischemia conditions, whereas 1539 and 2066 DEGs were revealed under Semax and ACTH(6–9)PGP peptides at 24 h after tMCAO. Furthermore, both peptides significantly reduced expression distortions caused by ischemia for 1171 genes associated with immune and neurosignaling pathways. Concomitantly, there were 32 DEGs under ACTH(6–9)PGP versus Semax administration at 24 h after tMCAO. Besides, neurogenesis-, angiogenesis-, protein kinase- and growth factor-related DEGs were revealed under peptides action. Previously, we observed the neuroprotective effect of peptides at the histological level in rat brains at 24 h after tMCAO. Thus, here we demonstrate the transcriptome manifestation of this histological effect. Furthermore, comparison with previous data at the 4.5 h post-tMCAO time point showed that the pattern of peptide action on the transcriptome depends on the time elapsed after tMCAO. **Conclusions:** We revealed that the effect of ACTH(6–9)PGP was more similar to Semax than different from it a day after tMCAO. At this time point, ACTH-like peptides compensated rat brain gene expression profiles disrupted by ischemia. Thus, our results may be useful for selecting more effective structures for future anti-stroke drugs and appropriate post-stroke time points for their testing.

## 1. Introduction

Ischemic strokes cause serious disturbances to the neurological functions of victims [1,2]. In 2020, the global prevalence of ischemic stroke was 68.16 million people [3]. In addition, stroke was one of the leading cause of disability-adjusted life years globally [4]. Strategies for the use of neuroprotection for the treatment of stroke have been actively developed [5]. Additionally, stroke therapy is extremely limited. One of the reasons is the lack of necessary drugs with known mechanisms of action. It was shown that the use of animal models, as well as the transcriptome analysis of brain cell activity under these conditions can help in drug mechanism discoveries. Interesting results were obtained for many substances, including peptides Sal-like 4 [6], TAT-STEP [7], NP1 [8], OxA [9], and others.

Various hormones are involved in ischemic and neurotrauma events. Moreover, the neuroendocrine system may play a decisive role in patients’ vulnerability and susceptibility to stroke [10,11,12]. Adrenocorticotropic hormone (ACTH) serves as the basis for the creation of synthetic neuroprotective agents [13,14,15]. In particular, natural ACTH(1-13) sequence was associated with a clear anti-inflammatory, neurogenic and neuroprotective effect in ischemic stroke models [16,17]. Besides, synthetic ACTH(4–7)PGP, which is known as Semax, has therapeutic effects but has no hormonal activities, development of drug dependence or withdrawal syndrome [18]. In Semax, the C-terminal tripeptide Pro-Gly-Pro (PGP) was designed to increase the duration of action of ACTH(4–7). Previously, the applicability of transcriptome analysis to the study of the effect of Semax on cerebral ischemia in model animals was demonstrated. Moreover, Semax partially reversed changes in gene expression that are affected by ischemia at 24 h after transient middle cerebral artery occlusion (tMCAO) in subcortical structures of rat brains containing an ischemic core. The genes of Semax-related patterns were grouped into a neurotransmitter cluster and an inflammatory clusters [19]. In addition, Semax affected the levels of some proteins in the ischemic brain of rats [20].

Recently, ACTH(6–9)PGP, another substance based on ACTH, was propose as a prospective drug. It should be noted that ACTH(6–9)PGP enhanced the viability of cultured cortical neurons [21]. In a model of Parkinson’s disease, peptides modulated the activity of genes associated with proliferation and survival (NF-kB and NRF2) [22]. Furthermore, at the transcriptomic level, we found partial overlapping of the effects of Semax and ACTH(6–9)PGP in the early (4.5 h) post-stroke period [23]. Previously, immunohistochemical, morphological, and morphometric study revealed that both peptides induce vascularization of brain tissues in the perifocal zones of the frontal cortex and proliferative activity of neuroglial cells at 24 h after tMCAO [24].

The aim of the current study was a comparative analysis of the effects of ACTH(6–9)PGP and Semax on the transcriptome of the frontal cortex region of rats, including viable cells, the perifocal zone and ischemic area, 24 h after tMCAO. An important task was also the search for genes involved in the protective effects identified in the rat brain at the histological level for both peptides during IR. We also hypothesized that the peptides might alter the expression of genes that, in particular, mediate the proliferation and angiogenesis in penumbra cells previously observed at the histological level in rat brains. As a result, using RNA-Seq, numerous DEGs were identified in the regions of the frontal cortex (FC) of the rat brain under the influence of ACTH(6–9)PGP and Semax at 24 h after tMCAO. Moreover, both ACTH-like peptides significantly reduced gene expression of IR-related disturbances associated with immune and neurosignaling pathways. In addition, under IR conditions, the studied peptides had a compensatory impact on the functioning of numerous genes associated with cell proliferation and differentiation, neurogenesis and angiogenesis. Concomitantly, there were peptide-specific effects on the transcriptome level. Furthermore, comparison with previous data at the 4.5 h post-tMCAO time point showed that the pattern of peptide action on the transcriptome depends on the time elapsed after tMCAO. Our results provide both similarity and difference between structural related peptides at the transcriptome level of ischemic brain cells a day after occlusion and may be useful for selecting more effective structures for future anti-stroke drugs.

## 2. Materials and Methods

### 2.1. Animals

White two-month-old male rats of the Wistar line that (weighted between 200 and 250 g) were provided by AlCondi, Ltd. (Moscow, Russia). The study animals were divided into four groups: “sham operation” (SO), “ischemia–reperfusion” (IR), “ischemia–reperfusion + Semax” (IS), and “ischemia–reperfusion + ACTH(6–9)PGP” (IA). Every experimental group included at least five animals.

### 2.2. Rat Transient Middle Cerebral Artery Occlusion (tMCAO) Model

The tMCAO model with 90 min of occlusion under isoflurane anesthesia was carried out according to the method of Koizumi et al. [25], as previously described [24]. The details of the tMCAO model are outlined in Supplementary Method S1. The decapitation procedure was performed on animals immediately after isoflurane anesthesia. The study rats were decapitated at 24 h after the beginning of occlusion/sham operation procedures.

The MRI study of ischemic injury in rat brains of IR, IS, and IA groups was carried out immediately before decapitation. Sham-operated (SO) rats were subjected to MRI on an equal basis with other rats. No rats from the SO group showed signs of ischemia according to the MRI data. Technical MRI details are outlined in Supplementary Method S2. ImageJ software (Wayne Rasband, National Institute of Mental Health, Bethesda, MD, USA) and Mann–Whitney U test calculation using Python (mannwhitneyu function from the scipy library of the Python) were used for quantitative assessment. The statistical difference between groups was significant at *p* < 0.05.

The rats of IS and IA groups were intraperitoneally injected with Semax and ACTH(6–9)PGP at 1.5, 2.5, and 6.5 h after surgery; the dosage of each peptide was 100 µg/kg of rat weight for each peptide, respectively, in accordance with the data from the literature [19,26,27]. Simultaneously, saline was injected into the study animals in both the IR and SO groups.

### 2.3. Collection of Samples and RNA Isolation

Ipsilateral fragments of the region of the frontal cortex (FC) were extracted within the range of +2 ± 0.5 to +5 ± 0.5 mm from the bregma. The resulting FC samples of the SO, IR, IS and IA groups were named SO24-f, IR24-f, IS24-f and IA24-f, respectively. Afterwards, the total RNA was isolated, and the integrity of the RNA was examined through capillary electrophoresis (Experion, BioRad, Hercules, CA, USA). The RNA integrity number (RIN) was at least 9.0.

### 2.4. RNA-Seq

An Illumina HiSeq 1500 instrument was used for RNA-Seq analysis to determine the polyA fraction of the total RNA, as previously mentioned [19]. The number of reads (1/50 nt) generated is at least 10 million. The RNA-Seq analysis was conducted with the participation of OOO Genoanalytika, Moscow, Russia.

### 2.5. cDNA Synthesis and Real-Time Reverse Transcription Polymerase Chain Reaction (RT-PCR)

Using oligo (dT)_18_ primers, cDNA synthesis was performed, as described in earlier works [19]. The PCR primers were selected using the OLIGO Primer Analysis Software version 6.31 and were synthesized by the Evrogen Joint Stock Company, Moscow, Russia (Supplementary Table S1). Using RT-PCR, each cDNA sample was analyzed three times, as previously mentioned [19].

### 2.6. RNA-Seq Data Analysis

In every comparison group, three animals (n = 3) were included (SO24-f, IR24-f, IS24-f, IA24-f) for RNA-Seq experiments. For genes annotations we used the Cuffdiff/Cufflinks software. The Cuffdiff program was used to measure mRNA expression levels, as described in previous works [19]. Only the genes that demonstrated >1.5-fold changes in expression and had *p*-values (*t*-test) adjusted using the Benjamini–Hochberg procedure below 0.05 (*Padj* < 0.05) were explored.

### 2.7. Real-Time RT-PCR Data Analysis

By using Relative Expression Software Tool (REST) 2005 software (gene-quantification, Freising-Weihenstephan, Bavaria, Germany) [28,29], the relative gene expression was calculated, as previously mentioned [19]. The calculation of values was based on Ef^Ct(ref)^/Ef^Ct(tar)^, where Ef is the PCR efficiency, Ct(tar) is the average threshold cycle (Ct) of the target gene, Ct(ref) is the average Ct of the reference gene, and Ef^Ct(ref)^ is the geometric average Ef^Ct^ of the reference genes. All PCR reactions had efficiency values within the range of 1.89 to 2.04 (Supplementary Table S1). The expression of cDNA samples was normalized using the *Gapdh* reference gene. Five animals were present in each comparison group. Significant differences were considered at *p* < 0.05.

### 2.8. Functional Analysis

The functions of the differentially expressed mRNAs (DEGs) were annotated by using the Database for Annotation, Visualization and Integrated Discovery (DAVID) (2021 Update) [30]. The functional categories that had *Padj* < 0.05 were the only ones that were considered. Hierarchical cluster analysis of the DEGs was carried out using Heatmapper (it was built using Shiny (version 0.12.2), a framework for R (version 3.2.0, Wishart Research Group, University of Alberta, Ottawa, ON, Canada) [31]. A volcano plot was built using Microsoft Excel (Microsoft Office 2010). Cytoscape 3.9.2 software (Institute for Systems Biology, Seattle, WA, USA) [32] was used for the visualization of the regulatory network.

### 2.9. Availability of the Data and the Material

The RNA-sequencing data was deposited in the Sequence Read Archive database under the access code PRJNA1119923 (SAMN41664920-SAMN41664931, https://dataview.ncbi.nlm.nih.gov/object/PRJNA1119923) (accessed on 25 June 2024) [33], as well as PRJNA916856 (SAMN32510043-SAMN32510057, http://www.ncbi.nlm.nih.gov/bioproject/916856) (accessed on 5 January 2023) [34].

## 3. Results

### 3.1. Magnetic Resonance Imaging (MRI)

According to the MRI data, the injury was formed in the ipsilateral hemisphere and spread to the subcortex and adjacent cortex at 24 h after tMCAO. Such injury localization was also typical for all rats after any peptide and saline administration at 24 h after tMCAO. Supplementary Figure S1 presents a quantitative assessment of the volume of injury. Data showed the differences between IR24-f, IS24-f and IA24-f groups. Additionally, typical diffusion-weighted imaging (DWI) with mapping of the apparent diffusion coefficient (ADC) scans of rat brains at 24 h after tMCAO is presented too. Such images indicate water movement within the tissue. Lesions with diffusion restriction appear bright on DWI scans and dark on ADC maps [35]. Using Mann–Whitney U test calculation, the Semax-treated group showed significant statistical difference compared with IR alone (*p* = 0.0159). Concomitantly, the ACTH(6–9)PGP-treated group did not show significant difference versus the IR group (*p* = 0.0556).

### 3.2. Effect of IR on the Gene Expression in FC of Rat Based on the RNA-Seq Data

**Figure 1:**
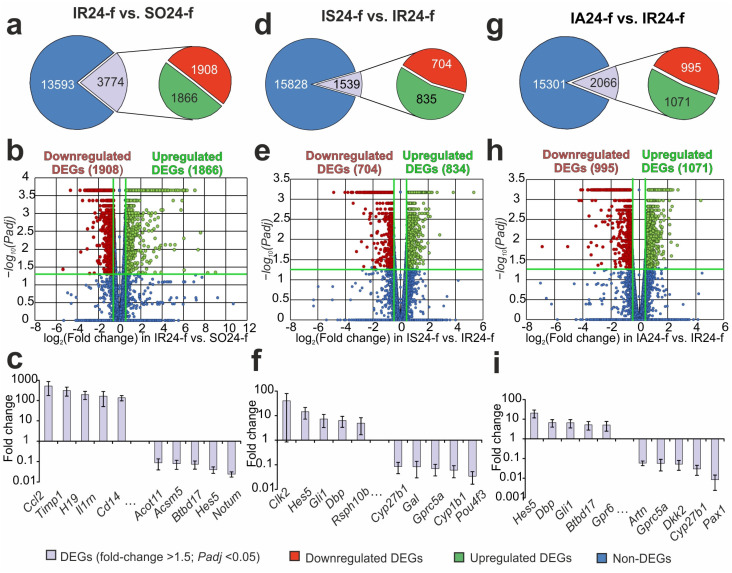
Effect of ischemia, ACTH(6–9)PGP and Semax on the transcriptome of FC of rats 24 h after tMCAO. (**a**,**d**,**g**) Results of RNA-Seq analysis for IR24-f vs. SO24-f (**a**), IS24-f vs. IR24-f (**d**), IA24-f vs. IR24-f (**g**). The quantity of DEGs is indicated by the numbers in the diagram sectors. (**b**,**e**,**h**) Volcano plots show a distribution data between the IR24-f and SO24-f (**b**), IS24-f and IR24-f (**e**), IA24-f and IR24-f (**h**) groups. (**c**,**f**,**i**) The top 10 DEGs (Fold change > 1.5; *Padj* < 0.05) with the highest expression changes in IR24-f vs. SO24-f (**c**), IS24-f vs. IR24-f (**f**), IA24-f vs. IR24-f (**i**). The data are presented as the mean ± standard error of the mean (M ± SEM).

Using RNA-Seq, we analyzed the transcriptional profile of the mRNAs for 17,367 genes in the FC of rats at 24 h after tMCAO. In a pairwise comparison of the RNA-Seq data for IR24-f vs. SO24-f (Figure 1a, Supplementary Table S2) we identified significant changes at the mRNA level for 3774 differentially expressed genes (DEGs) with 1866 up- and 1908 downregulated genes. The volcano plot in Figure 1b demonstrates the variations in mRNA expression levels between the IR24-f and SO24-f comparison groups. We noticed that the top five genes with increased expression level in response to IR were increased by ≥100 times, including *Ccl2*, *Timp1*, *H19*, *Il1rn* and *Cd14*. At the same time the top five DEGs with decreased expression level were *Acot11*, *Acsm5*, *Btbd17*, *Hes5* and *Notum* (Figure 1c).

Real-time PCR analysis of seven DEGs’ (*Nos3*, *Il4r*, *Jun*, *Vegfa*, *Gabrg2*, *Rasgrp2* and *Hes5*) expression patterns was used to study the effects in IR24-f vs. SO24-f pairwise comparisons to test the results of RNA-Seq on an extended sample of animals. Supplementary Figure S2 showed RNA-Seq and PCR results side by side on a diagram. Thus, RNA-Seq data were adequately verified using real-time PCR.

### 3.3. Effect of Semax and ACTH(6–9)PGP on the Transcriptome in FC of Ischemic Rats Based on the RNA-Seq Data

By using RNA-Seq, we revealed 1539 DEGs (835 up- and 704 downregulated DEGs) at 24 h after IR and Semax administration compared to the samples of IR24-f (Figure 1d,e, Supplementary Table S3). The volcano plot in Figure 1e shows the differences in mRNA expression levels between the IS24-f and IR24-f group of genes. The top five genes with increased expression level (*Clk2*, *Hes5*, *Gli1*, *Dbp* and *Rsph10b*) and with decreased expression level genes were *Cyp27b1*, *Gal*, *Gprc5a*, *Cyp1b1* and *Pou4f3*. They showed in Figure 1f.

Meanwhile, in the FC of rats who underwent IR after ACTH(6–9)PGP injections, we revealed 2066 DEGs (1071 up- and 995 downregulated) compared to saline-treated IR groups (IA24-f vs. IR24-f) (Figure 1g,h, Supplementary Table S4). The volcano plot in Figure 1h illustrates the differences in mRNA expression levels between the IA24-f and IR24-f group of genes. The top five upregulated DEGs in IA24-f vs. IR24-f pairwise comparisons were *Hes5*, *Dbp*, *Gli1*, *Btbd17* and *Gpr6*, whereas the top five most considerably downregulated genes were *Artn*, *Gprc5a*, *Dkk2*, *Cyp27b1* and *Pax1* (Figure 1i). In addition, there has been an increase in the expression level of multiple genes in both Semax and ACTH(6–9)PGP administration groups of upregulated (*Hes5*, *Gli1* and *Dbp*) and highly downregulated (*Cyp27b1* and *Gprc5a*) DEGs.

### 3.4. Comparison of RNA-Seq Results in Different Pairwise Comparisons in FC at 24 h After tMCAO

**Figure 2:**
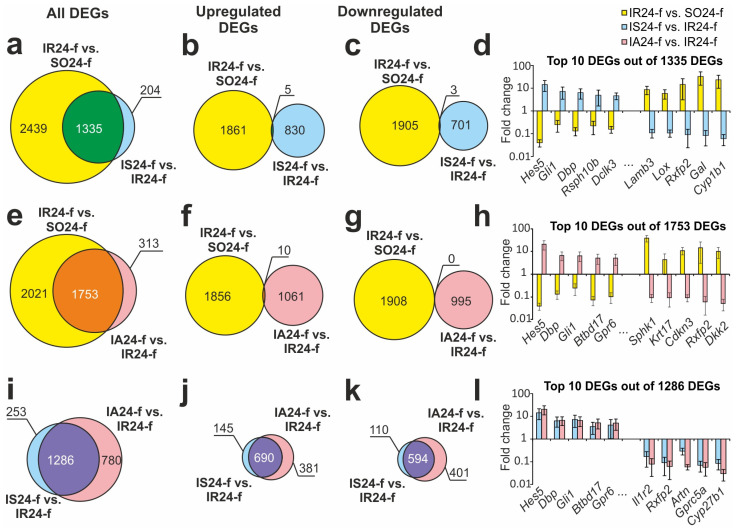
The gene expression changes for different groups of ischemia, ACTH(6–9)PGP and Semax at 24 h after tMCAO. Venn diagrams represent RNA-Seq results obtained in comparisons between the IR24-f vs. SO24-f (**a**–**c**); IS24-f vs. IR24-f (**e**–**g**); IA24-f vs. IR24-f (**i**–**k**) groups in FC. All (**a**,**e**,**i**), upregulated (**b**,**f**,**j**), and downregulated (**c**,**g**,**k**) DEGs are shown. The top 10 DEGs (Fold change > 1.5; *Padj* < 0.05) among 1335 (**d**), 1753 (**h**) and 1286 (**l**) in the Venn diagram (**d**,**h**,**l**), respectively, featuring the highest fold changes in the IR24-f vs. SO24-f (**d**); IS24-f vs. IR24-f (**h**); IA24-f vs. IR24-f (**l**) comparison groups. The data are presented as M ± SEM.

We revealed that both ACTH(6–9)PGP and Semax peptides changed the IR-related gene expression pattern. Hence, we revealed 1335 overlapping DEGs in the IS24-f vs. IR24-f and IR24-f vs. SO24-f groups (Figure 2a, Supplementary Table S5). Comparison between only upregulated genes and only downregulated genes under both conditions are presented in the form of Venn diagrams in Figure 2b,c. We revealed only five upregulated DEGs (*Angptl4*, *Ssc5d*, *Fcgr3a*, *Irf8*, and *Anxa3*) and three downregulated DEGs (*Slc13a4*, *Arc*, and *Nr4a1*) that co-directly changed their mRNA level in both cases (Figure 2b,c). Hierarchical cluster analysis of such DEGs in the comparison groups is presented in Supplementary Figure S3. Figure 2d presented the top 10 overlapping DEGs that had the highest expression changes in IS24-f vs. IR24-f. As can be seen, the Semax peptide diminished the effect of ischemia on their expression.

We also found 1753 overlapping DEGs in the IA24-f vs. IR24-f and IR24-f vs. SO24-f groups (Figure 2e, Supplementary Table S6). Venn diagrams with only upregulated genes and only downregulated genes under both conditions are presented in Figure 2f,g. Interestingly, we did not find any downregulated DEGs, meanwhile there were ten upregulated DEGs (*Mcm3*, *Itgal*, *Plac8*, *S100a4*, *Ifi27l2b*, *Ssc5d*, *Fcgr3a*, *Mx1*, *Irf8*, and *Fgfrl1*), both under IR and ACTH(6–9)PGP action (Figure 2f,g). Hierarchical cluster analysis of such DEGs in pairwise comparisons is presented in Supplementary Figure S3. The top 10 overlapping DEGs that had the greatest fold change in IA24-f vs. IR24-f are shown in Figure 2h. As can be seen, the ACTH(6–9)PGP also diminished the effect of IR on their expression level.

We revealed that 780 and 253 DEGs were unique for the ACTH(6–9)PGP and Semax action, after a comparison of the RNA-Seq results of the IS24-f vs. IR24-f and IA24-f vs. IR24-f groups, respectively. However, 1286 DEGs were overlapped between both peptides action at 24 h after tMCAO (Figure 2i, Supplementary Table S7). Interestingly, almost all DEGs co-directionally changed their expression level under both peptides after IR. In particular, 690 DEGs were up- (Figure 2j) and 594 DEGs were downregulated (Figure 2k), both in IS24-f vs. IR24-f and IA24-f vs. IR24-f pairwise comparisons. The exception was the *Angptl4* gene encoded angiopoietin-like 4 and *Npas4* gene encoded neuronal PAS domain protein 4. Peptides had different effects on their differential expression in FC under IR. Figure 2l shows the top 10 overlapping genes with the greatest fold change in the IA24-f vs. IR24-f groups. As can be seen, both peptides had a similar effect on the differential expression of these genes.

Thus, both ACTH(6–9)PGP and Semax peptides predominantly initiated a gene expression response that reduced expression disturbances caused by ischemia. So, those genes that were upregulated by IR, decreased their expression level under the influence of peptides on the contrary, as well as those genes that were downregulated by IR, increased their expression level under the influence of peptides predominantly.

**Figure 3:**
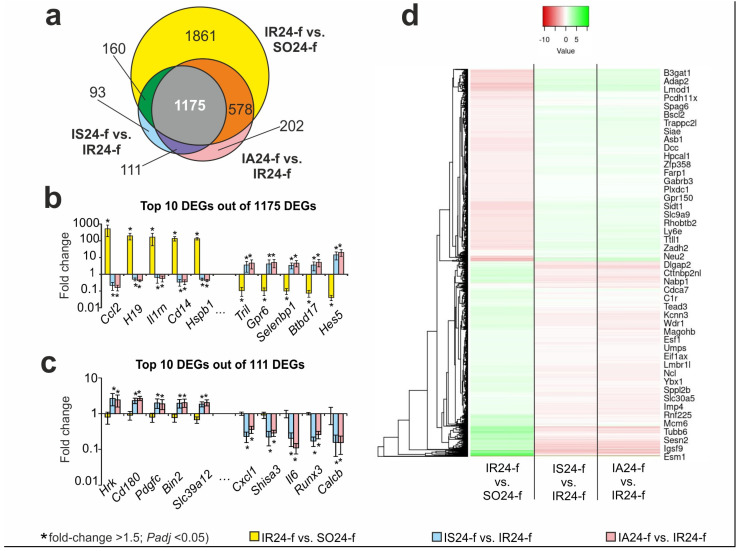
The RNA-Seq results for the IR24-f vs. SO24-f, IS24-f vs. IR24-f, IA24-f vs. IR24-f and IA24-f vs. IS24-f pairwise comparisons. (**a**) Venn diagrams present comparison of results between the IR24-f vs. SO24-f, IS24-f vs. IR24-f and IA24-f vs. IR24-f groups. The top 10 DEGs that lie within the intersection between the gene sets in the Venn diagram (**a**) and have the highest fold change in IR24-f vs. SO24-f (**b**). The top 10 DEGs that lie within the gene sets in the Venn diagram (**a**) among the overlapping section between IS24-f vs. IR24-f and IA24-f vs. IR24-f but not in the IR24-f vs. SO24-f pairwise comparisons (**c**). The data are presented as M ± SEM. (**d**) Hierarchical cluster analysis of all DEGs in IR24-f vs. SO24-f, IS24-f vs. IR24-f, IA24-f vs. IR24-f, where each row represents a DEG; n = 3 per group. Only those genes with cut-off >1.5 and *Padj* < 0.05 were selected recognized by significant.

The results of the triple comparison (IR24-f vs. SO24-f, IS24-f vs. IR24-f, and IA24-f vs. IR24-f. The results of the triple comparison (IR24-f vs. SO24-f, IS24-f vs. IR24-f, and IA24-f vs. IR24-f) are presented in a Venn diagram (Figure 3a). We identified 1175 genes that were DEGs by all the conditions. Both ACTH(6–9)PGP and Semax treatment initiated reverse changes in the expression of 1171 out of 1175 overlapped DEGs compared to the effects of IR (Supplementary Table S8). The top ten of them included the *Ccl2*, *H19*, *Il1rn*, *Cd14*, *Hspb1*, *Tril*, *Gpr6*, *Selenbp1*, *Btbd17*, and *Hes5* genes and are shown in Figure 3b. Additionally, the triple comparison made it possible to value relative complements on the Venn diagram. So, there were genes whose expression level changed only under the impact of one of the influencing factors. Thus, the IR factor only, without any peptides, caused 1861 DEGs (*Hspa1*, *Ccl4*, *Ptges*, *Stc2*, *Ptgir*, etc.). Moreover, in the IS24-f vs. IR24-f relative complement, there were 93 DEGs (*Clk2*, *Tnfrsf25*, *Map3k1*, *Grik4*, *Ccl6*, etc.). Their expression profile was specifically modulated by Semax but not the ACTH(6–9)PGP peptide at 24 h after occlusion. Concomitantly, there were 202 DEGs (*S100a9*, *Tnfsf12*, *Cplx3*, *Hspa13*, *Cxcl2*, etc.) that lay only in the IA24-f vs. IR24-f relative complement of the Venn diagram. Thus, ACTH(6–9)PGP, but not Semax, specifically caused their differential expression profile at 24 h after occlusion. Interestingly, there were 111 DEGs that overlapped between both peptides action but were non-DEGs under IR action. This gene set included *Hrk*, *Cd180*, *Tfap4*, *Pdgfc*, *Ly86*, *Lif*, *Il11*, *Serpine1*, *Cyp27b1*, and *Gprc5a* genes (Figure 3c, Supplementary Table S9). The peptides caused similar changes in the expression profile of these genes in FC at 24 h after tMCAO. The gene expression profile shows that, in addition to the general compensatory effect, the peptides have an effect on genomic activity that is not overlapping with ischemia action.

Figure 3d illustrated cluster analysis of all the DEGs in the IR24-f vs. SO24-f, IS24-f vs. IR24-f, and IA24-f vs. IR24-f in the form of a heat map. Both peptides significantly reduced gene expression activated by IR and increased expression reduced by IR. At the same time, a differential expression profile of genes is observed, associated with the unique effect of two peptides on genomic activity in FC at 24 h after tMCAO conditions.

### 3.5. Functional Enrichment Analysis of DEGs Altered in Different Comparison Groups

**Figure 4:**
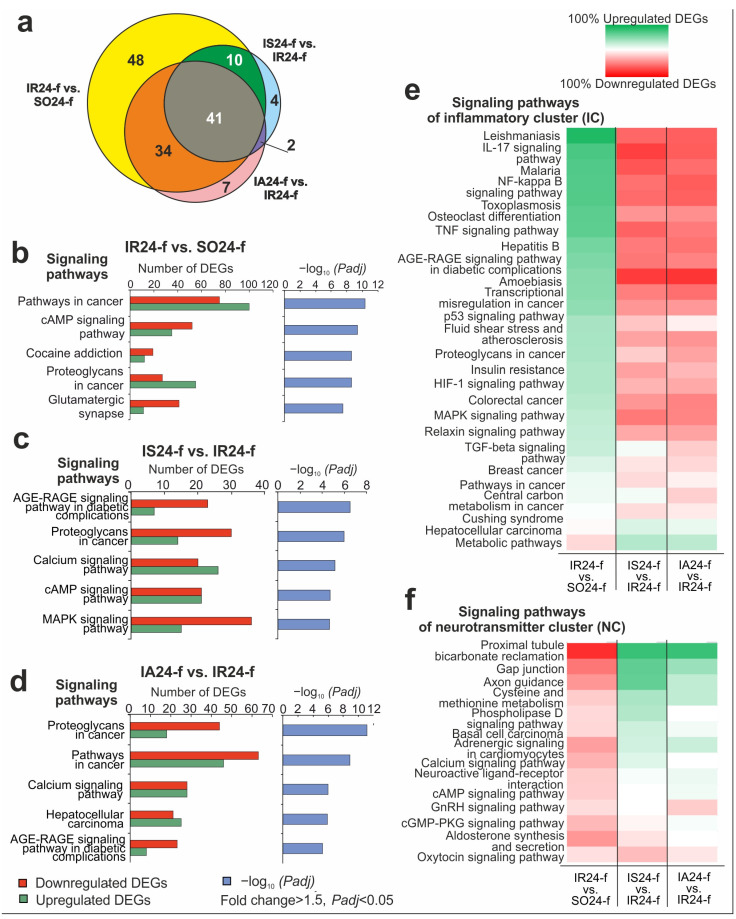
Signaling KEGG pathways associated with DEGs in the FC of rats 24 h after occlusion. DAVID (2021 Update) was used for pathway search in the IR24-f vs. SO24-f, IS24-f vs. IR24-f, IA24-f vs. IR24-f comparisons. (**a**) 3-set Venn diagram presents a comparison of DEG-related pathways in the IR24-f vs. SO24-f, IS24-f vs. IR24-f, IA24-f vs. IR24-f and IA24-f vs. IS24-f groups. The number on the chart segments indicates the numbers of annotations. (**b–d**) The most significant pathways in IR24-f vs. SO24-f (**b**), IS24-f vs. IR24-f (**c**), IA24-f vs. IR24-f (**d**) are presented. (**e**,**f**) Cluster analysis of overlapped signaling pathways associated with DEGs in IR24-f vs. SO24-f, IS24-f vs. IR24-f, IA24-f vs. IR24-f pairwise comparison. Pathways of inflammatory cluster (IC) (**e**) and neurotransmitter cluster (NC) (**f**) are presented. Each column represents a pairwise comparison and each row represents a signaling pathway (KEGG). The green bars represent the pathways with which the majority of upregulated genes are associated, and the red bars represent the pathways with which the majority of downregulated genes are associated. Only DEGs and pathways with *Padj* < 0.05 were selected as significant, n = 3 of animals per group.

Using DAVID v.2021, a KEGG pathway analysis of DEGs in different comparison groups was carried out. As a result, 133, 57 and 84 KEGG pathways were identified with *Padj* < 0.05 for DEGs of IR24-f vs. SO24-f, IS24-f vs. IR24-f, IA24-f vs. IR24-f, respectively. We compared KEGG annotations to found overlapping and specific data between IR24-f vs. SO24-f, IS24-f vs. IR24-f, and IA24-f vs. IR24-f pairwise comparisons. Results are illustrated using a 3-set Venn diagram in Figure 4a (Supplementary Table S10). Simultaneously, 11 pathways were overlapped between IR24-f vs. SO24-f and IS24-f vs. IR24-f, and 34 pathways were overlapped between the IR24-f vs. SO24-f and IA24-f vs. IR24-f pairwise comparisons. Remarkably, there were 7 and 43 overlapping pathways between IS24-f vs. IR24-f and IA24-f vs. IR24-f and between triple IR24-f vs. SO24-f, IS24-f vs. IR24-f and IA24-f vs. IR24-f, respectively. The top five pathways with the most significant *Padj* value in IR24-f vs. SO24-f included a cAMP signaling pathway and glutamatergic synapse (Figure 4b). Predominantly downregulated genes were associated with such pathways. Nevertheless, DEGs associated with pathways of inflammation (signaling pathways of proteoglycans, TNF, MAPK, p53, Osteoclast differentiation) mostly upregulated the expression levels. It is important to note that the MAPK and proteoglycan signaling pathways were included in the top five of the Semax-related list (Figure 4c). In addition, predominantly downregulated genes were also associated with such pathways in IS24-f vs. IR24-f. ACTH(6–9)PGP, and also predominantly decreased the expression level of the genes associated with the pathways of proteoglycan, MAPK, as well as NF-kappa B, IL-17, TNF, and other signaling pathways of inflammatory response (Figure 4d, Supplementary Table S10). Thus, DEGs and pathways associated with the action of peptides formed an inflammatory cluster (IC). These effects are the opposite in cases of ischemia, indicating the anti-inflammatory action of peptides (Figure 4e). Additionally, there were some genes of IC, including *Prkra*, *Sema4g*, *Aif1*, *Cxcl12*, which increased expression level under both peptides. Besides, among IC, the Irf5 gene was found for Semax only and *Ackr2*, *Ifi35*, *Igsf1*, *Ifi27l2b*, *Cx3cl1*, *Aif1l*, *Sema4b* genes were found for ACTH(6–9)PGP only as upregulated DEGs versus saline administration at 24 h after tMCAO (Supplementary Table S11).

Concomitantly, pathways of neuroactive ligand-receptor interaction, cAMP, Axon guidance, Cysteine and methionine were associated with mostly upregulated genes both under Semax and ACTH(6–9)PGP action. Thus, DEGs and pathways associated with the action of peptides formed a neurotransmitter cluster (NC). These effects are the opposite in cases of ischemia, indicating the neuroprotective action of peptides (Figure 4f, Supplementary Table S10). Additionally, there were some genes of NC associated with the oxytocin signaling pathway. Their list included *Pla2g4b*, *Mylk*, *Rgs2*, *Cdkn1a*, *Adcy4*, *Jun*, *Cacng4*, *Nos3*, *Ptgs2*, *Prkab2*, *Kcnj12*, which were downregulated both under Semax and ACTH(6–9)PGP action. Concomitantly, those genes were downregulated under IR conditions and vice versa. Thus, results indicate almost complete opposition of effects in ischemia versus peptide administration, indicating decreased negative ischemia effects at 24 after tMCAO in penumbra-associated cells of FC.

Additionally, using RNA-Seq, we found a number angiogenesis-, neurogenesis- and growth factor-related DEGs in FC under peptides action. Most of them were subjected to the compensatory effect of peptides under IR conditions. Moreover, genes encoded with growth factors (*Hgf*, *Igfbp5*, *Pdgfra*, *Pdgfc*, *Gdf11*, *Grb14*), factors of neuronal differentiation (*Neurod1*, *Neurod6*, *Neurod2*), protein kinases and related proteins (*Map2k6*, *Srpk3*, *Prkra*, *Mapk3*, *Mok*, *Camk2g*, *Mapk1*, *Prkar2b*, *Hipk4*, *Map2k5*) were upregulated after administration any Semax and ACTH(6–9)PGP compared to saline under IR conditions (Supplementary Table S12).

### 3.6. The Search for Pathways and Genes That Reflect Gene Expression Effects of ACTH(6–9)PGP and Semax Peptides a Day After IR

**Figure 5:**
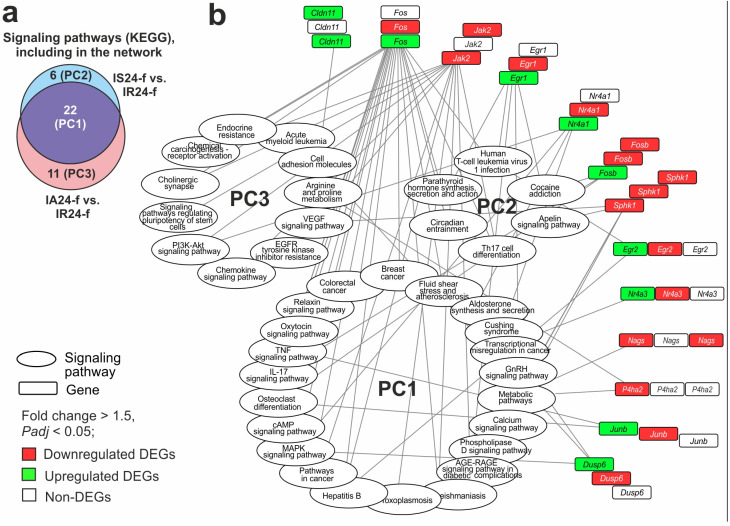
The search for pathways that reflect gene expression effects of Semax and ACTH(6–9)PGP at 24 h after tMCAO in FC. (**a**) Venn diagram presents overlapped and unique DEG-related annotations in the IS24-f vs. IR24-f and IA24-f vs. IR24-f pairwise comparisons. Only pathways that have annotations with DEGs in IA24-f vs. IS24-f pairwise comparison are included. The number on the chart segments indicates the numbers of annotations. All pathways were grouped on three pathway clusters (PC1, PC2 and PC3) (**b**) The gene network of effects of ACTH(6–9)PGP and Semax peptides on transcriptome of FC at 24 h after tMCAO. In the scheme, 13 genes that are DEGs in the IA24-f vs. IS24-f are presented. These genes are grouped by three half rings of rectangles colored according to their differential expression in comparison groups. Each half ring includes the same 32 genes, but the color in the inner half ring indicates DEGs in the IA24-f vs. IS24-f, the color in the central half ring indicates DEGs in the IS24-f vs. IR24-f, and the colour in the outer half ring indicates the DEGs in IA24-f vs. IR24-f. Three pathway clusters (PC1, PC2 and PC3) are grouped in ovals. PC1 represents common pathways that lie within the intersection between the IA24-f vs. IR24-f and IS24-f vs. IR24-f pathway sets in the Venn diagram (Figure 4a) and reflects effects of both peptides. PC2 represents unique pathways that lie within the pathway sets among the IS24-f vs. IR24-f but not among the IA24-f vs. IR24-f pairwise comparisons in the Venn diagram (Figure 4a). PC3 represents unique pathways that lie within the pathway sets among the IA24-f vs. IR24-f but not among the IS24-f vs. IR24-f pairwise comparisons in the Venn diagram (Figure 4a). The lines connecting the genes and pathways indicate association between them. The KEGG databases from DAVID v. 2021 were used to annotate all clustered pathways. Only DEGs and pathways with *Padj* < 0.05 were selected as significant. The network was constructed using Cytoscape 3.9.2.

We identified 98 peptide-related pathways in total for the more than two thousand DEGs altered under ACTH(6–9)PGP and Semax action in IS24-f vs. IR24-f and IA24-f vs. IR24-f pairwise comparisons (Supplementary Table S10). Among them, 43 pathways overlapped, whereas 14 and 41 pathways characterized the specific effects of Semax and ACTH(6–9)PGP under IR, respectively. These results were obtained regarding the effect of IR by the superimposing of peptides action data in two IS24-f vs. IR24-f and IA24-f vs. IR24-f pairwise comparisons (Venn diagram method). However, in order to better understand which genes and pathways the peptides act on, another method of data comparison should be used. It is based on a direct comparison of the data in the ACTH(6–9)PGP group relative to the Semax group. As a result, we found 32 DEGs (14 up- and 18 downregulated) in IA24-f vs. IS24-f that marked the different effects of peptides on the transcriptome (Supplementary Table S13). We revealed that 13 out of 32 DEGs (*Nags*, *P4ha2*, *Sphk1*, *Jak2*, *Fos*, *Nr4a1*, *Dusp6*, *Egr1*, *Fosb*, *Junb*, *Cldn11*, *Egr2*, *Nr4a3*) had associations with 39 out of 98 peptide-related pathways. A total of 22 pathways overlapped between IA24-f vs. IR24-f and IS24-f vs. IR24-f pairwise comparisons, whereas 11 and 6 of them lay in relative complements for ACTH(6–9)PGP and Semax, respectively (Figure 5a). Thus, pathways were grouped on three pathway clusters (PC), namely PC1, PC2 and PC3, respectively. Figure 5b shows the network between genes and the pathways involving the protein products of these genes. The network contained 13 genes, 22 pathways of PC1, 6 pathways of PC2 and 11 pathways of PC3 in summary. In the network, the genes are shown by three half rings of blocks of the same genes. However, the inner half ring identifies the DEGs in the IA24-f vs. IS24-f, the central half ring identifies DEGs in the IS24-f vs. IR24-f, and the outer half ring determines the DEGs in IA24-f vs. IR24-f. Three pathway clusters (PC1, PC2 and PC3) are grouped in the ovals. The lines connecting the genes and pathways indicate association between them. It should be noted that associations of genes were revealed for pathways from each PC1, PC2 and PC3. Almost all genes were overlapped between DEGs in IS24-f vs. IR24-f and IA24-f vs. IR24-f pairwise comparisons, whereas only one P4ha2 gene encoding prolyl 4-hydroxylase, alpha polypeptide II, was a unique DEG for IA24-f vs. IS24-f (Figure 5b).

PC1 included common pathways identified both in IA24-f vs. IR24-f and IS24-f vs. IR24-f (Figure 5b). Among the PC1 pathways were Calcium, Oxytocin, MAPK, cAMP, IL-17, GnRH and other signaling pathways. Ten genes (*Fos*, *Nr4a1*, *Egr1*, *Fosb*, *Junb*, *Egr2*, *Sphk1*, *Jak2*, *Nags*, *P4ha2*) were involved in the presentation of common pathways in the network. Each of these genes changed expression in IA24-f vs. IS24-f. So, ACTH(6–9)PGP can had a significantly altered effect than Semax on expression regulation within overlapping pathways due to the different impact on the expression of these genes. It should be noted that three genes (*Nags*, *Junb* and *Nr4a3*) had connecting lines only within the pathways in PC1.

PC2 included pathways identified in IS24-f vs. IR24-f, but not among IA24-f vs. IR24-f (Figure 5b). Among the PC2 pathways were Th17 cell differentiation and Human T-cell leukemia virus 1 infection, as well as Parathyroid hormone synthesis, secretion and action, Apelin and other signaling pathways. Six genes (*Sphk1*, *Jak2*, *Fos*, *Egr1*, *Fosb*, *Egr2*) were involved in the presentation of Semax-specific pathways among DEGs in IA24-f vs. IS24-f. It should be noted that *Fos*, *Egr1* and *Egr2* genes changed their expression only under Semax action versus saline (IS24-f vs. IR24-f), whereas on expression of *Fosb* gene in this comparison Semax had a significantly greatest effect than ACTH(6–9)PGP versus saline (IA24-f vs. IR24-f).

Finally, we formed PC3 that included Cell adhesion molecules, Chemokine, VEGF, PI3K-Akt, Cholinergic synapse and other pathways (Figure 5b). The PC3 included pathways identified in IA24-f vs. IR24-f but not among the IS24-f vs. IR24-f. Seven DEGs in IA24-f vs. IS24-f (*P4ha2*, *Sphk1*, *Jak2*, *Fos*, *Nr4a1*, *Dusp6*, *Cldn11*) were involved in the presentation of ACTH(6–9)PGP-specific pathways. Particularly, *Jak2* and *Cldn11* genes change expression only under ACTH(6–9)PGP but not Semax versus saline in IA24-f vs. IR24-f. Furthermore, *Jak2* gene was associated with the largest number (6) of pathways of PC3. Concomitantly, *Cldn11* gene was alone that had connecting lines only within the pathways in PC3.

Thus, the network (Figure 5b) reflected gene expression effects of ACTH(6–9)PGP and Semax peptides on transcriptome of FC at 24 h after tMCAO.

## 4. Discussion

In the present study, the IR- and peptide-related effects on the transcriptome at 24 h after tMCAO in rat brains were studied. The MRI of all ischemic rats detected the ischemic lesion with hemispheric localization after tMCAO. Addtionally, the Semax-treated group showed significant statistical difference in volume of injury compared with IR alone. Previously, we showed that, under the conditions of the tMCAO model used, the region of the frontal cortex (FC) of the ipsilateral hemisphere contained not only ischemic tissue, but also penumbra and viable cells [24]. In this brain region, we assessed the genome-wide mRNA pattern at 24 h after tMCAO, as well as ACTH(6–9)PGP and Semax action. Using RNA-Seq analysis, 1000 DEGs were identified after IR and peptides at 24 h after tMCAO in FC cells. Additionally, both the RNA-Seq and the RT-PCR methods showed a similar differential expression pattern for the set of tested genes.

The RNA-Seq results showed that many genes of immunity and inflammation mainly increase their mRNA level after IR in penumbra-associated FC cells (Figure 4e). This is consistent with numerous previous studies and reviews showing activation of inflammation at the cellular and transcriptional level within an ischemic injury [36,37,38,39,40]. According to our data, genes associated with neurosignaling pathways were predominantly downregulated in response to IR (Figure 4e). Among them were genes and encoding proteins of a whole series of cell receptor systems, including glutamatergic, cholinergic, GABAergic etc. (Figure 4f, Supplementary Table S10). The suppression of the neurotransmission under conditions of brain ischemia has been noted previously [41,42,43,44]. In addition, the involvement of these receptor systems in the mechanism of the neuroprotective action of a number of compounds has been shown [45,46,47,48,49,50]. Previously, we revealed more than 1000 DEGs in the subcortex of rat brains, where ischemic focus was localized at 24 h after tMCAO [51,52]. Among them there were two major clusters of genes that reproduced their genetic profile both in subcortical structures and FC a day after IR. So, inflammatory cluster (IC) amounted to *Hspa1a*, *Socs3*, *Hspb1*, *Cd14*, *Cd44*, *Il1rn* and other genes that were upregulated under IR conditions. Additionally, the neurotransmitter cluster (NC) amounted to *Adra2c*, *Chrm1*, *Grm3*, *Grin2a*, *Chrna7*, *Drd1*, *Gabra5* and other genes that were downregulated under IR conditions in both tissues. Concomitantly, there were some IC genes, including *Tll1*, *Ccl11*, and *RT1-M6-2*, as well as some NC genes, including *Htr1a*, *Hrh2*, and *Grin2c*, that were DEGs in FC but not in subcortical structures at 24 h after tMCAO. The result may indicate a heterogeneous profile of genetic inflammatory and neurotransmitter response in different brain regions at 24 h after tMCAO.

Here, we confirmed the compensatory influence of Semax on the functioning of a large number of genes under IR conditions. Previously, we revealed that Semax action aimed at reduced gene expression disturbances caused by ischemia at 24 h after tMCAO in subcortical regions with an ischemic core. So, downregulation of expression was identified for genes of IC and upregulation of expression was identified for genes of NC [19]. Here, the Semax peptide administrations also led to a decrease in the expression of IC genes and increase in the expression of NC genes in FC at 24 h after tMCAO. A number of IC genes, including *Cd14*, *Hspb1*, *Socs3*, *Cd44*, *Tnfrsf12a*, *Cxcl16*, *RT1-Da*, *Cd74* and NC genes, including *Chrm1*, *Neu4*, *Neurod6*, *Grm3*, *Grm5*, *Drd1*, *Drd2* overlapped between FC and subcortical structures DEG sets under Semax action. Concomitantly, we observed some differences in the spectrum of DEGs between two brain parts. So, *Tgfb1*, *Cd63*, *Hif1a*, *RT1-A2*, *RT1-M6-2* and other genes of IC were DEGs only in FC. Additionally, *Adra1d*, *Npy5r*, *Grin2c*, *Chrnb2*, *Grik2* and other NC genes were also DEGs only in FC under Semax action. Such a difference, on the one hand, may be caused by objective differences in the of tMCAO implementation. Our result can also indicate that the Semax effect depends on the transcriptomic profile caused by IR conditions in the different brain samples at different distances from the focus of ischemia. It should also be noted that in the FC there were more genes whose profile was compensated for in contrast to subcortical structures under Semax action (1323 DEGs vs. 312 DEGs). This may be due to the large number of cells capable of recovery in the area of the penumbra and healthy tissue, in contrast to the area of the ischemic focus, respectively. This example provides additional evidence that the transcriptomic response of Semax action correlates with the conditions in which the brain cells are exposed to ischemia, depending both on the characteristics of the stroke model and on the damage in the brain region being studied.

In this paper, in addition to Semax, we studied the transcriptomic effects of the ACTH(6–9)PGP peptide in the FC at 24 h after tMCAO. Prior to the current study, we demonstrated the effect of this peptide under conditions of cerebral ischemia at 4.5 h after tMCAO [23]. Undoubtedly, each of the peptides had some unique features due to the difference in the structural elements of ACTH. Nevertheless, in each case, the effect of the ACTH(6–9)PGP peptide was mostly close to the Semax effect. Here, both the ACTH(6–9)PGP and Semax peptide administrations led to a decrease in the expression of IC genes and increase in the expression of NC genes in FC at 24 h after tMCAO. Both peptides showed similar effects aimed at reduced expression disturbances caused by ischemia. For instance, such effects were for *Cd14*, *Hspb1*, *Socs3*, *Cd44*, *Ccr1*, *RT1-M6-2* and other IC genes and *Adra1d*, *Npy5r*, *Grin2c*, *Chrnb2*, *Grik2*, *Grm3* and other NC genes. It should be noted that more than 1000 genes were identified as common between the ACTH(6–9)PGP and Semax peptides action. It is possible that overlapped genes form the core of the genetic neuroprotection potential necessary for recovery processes after a stroke in FC.

When trying to compare ACTH(6–9)PGP and Semax’s impact on the transcriptomic profile of FC cells, we revealed hundreds of genes that lie in relative complement for each peptide under IR, in accordance with the Venn diagram method (Figure 3a). Thus, specific gene sets identified were associated with functional specific annotations of peptide effects (Figure 4a). Another method of data comparison that gave us a better understanding of which genes and pathways the peptides act on differently was based on a direct comparison of the data in the ACTH(6–9)PGP group relative to the Semax group. As a result, there were only 32 genes that changed expression levels (*Padj* < 0.05) in the ACTH(6–9)PGP versus Semax comparison under IR conditions (Supplementary Table S13). These genes had associations with three pathway clusters (PC1-PC3). PC1 united pathways that were associated with both the action of ACTH(6–9)PGP and Semax. PC2 united pathways associated only with the action of Semax. PC3 was associated only with the action of ACTH(6–9)PGP. Pathways of IL-17, TNF, MAPK and other for inflammatory response, as well as cAMP, Oxytocin, Calcium and others for neurotransmitter response were included in PC1 that reflected an overlapped peptide-induced gene expression profile. T-cell-related pathways were specific for Semax action predominantly, whereas PI3K-Akt, VEGF, cholinergic synapse pathways were specific for ACTH(6–9)PGP action under IR. For the *Sphk1* gene that encoded sphingosine kinase 1 enzyme and *Fosb* gene that encoded G0/G1 switch regulatory protein 3, we found an association with the signaling pathways of PC1-PC3. It is known that these genes are involved in the regulation of the ischemic process, including neuroinflammation [53,54,55]. Additionally, the *Angptl4*, and *Npas4* genes were characterized by differently directed changes in expression under the influence of the ACTH(6–9)PGP and Semax peptides. So, *Npas4* gene was downregulated under ACTH(6–9)PGP and upregulated under Semax action (Supplementary Table S13). Unlike *Npas4*, *Angptl4* gene was upregulated under ACTH(6–9)PGP and downregulated under Semax action, vice versa (Supplementary Table S13). Differential expression of the Janus kinase 2 (*Jak2*) gene was significantly different in the ACTH(6–9)PGP group from the Semax group and was reduced under ACTH(6–9)PGP action. This gene was associated with the maximum number of pathways included in PC1-PC3. Additionally, *Fos* gene was multiple hub for the pathways of PC1-PC3. Its expression level was increased under ACTH(6–9)PGP versus Semax administration and also characterized differences between peptides actions in IR conditions. In addition, Fos is involved in the mechanisms of neuroprotection under the influence of drugs [56]. Previously, we showed downregulation of the Fos gene both at the mRNA and protein level in the frontoparietal cortex adjacent to ischemic focus at 24 h after tMCAO under Semax action [20]. The involvement of *Npas4*, *Angptl4*, *Jak2* and *Fos* genes in cerebrovascular pathologies has been shown previously [57,58,59,60,61].

In total, we revealed that the effect of ACTH(6–9)PGP was more similar to Semax than different from it at 24 h after tMCAO. Nevertheless, when peptides act on cells under different conditions, the ratio of general and unique effects of peptides may also be different. Previously, we revealed DEGs at 4.5 h after tMCAO in FC under both peptides action. Comparisons of DEG sets between the two time points after ischemia are presented in the Supplementary Figure S3. Previously, we observed, after 4.5 h, some of the genes encoding cytokines, cell adhesion molecules and other inflammatory proteins were upregulated under the action of Semax rather than ACTH(6–9)PGP at 4.5 h after tMCAO, whereas IR also increased expression of these genes [23]. Here we found only single genes that showed enhancing effects of at least one of the peptides on the effect of ischemia itself. Particularly, genes of IC were upregulated, and genes of NC were downregulated under both IR and Semax action (Supplementary Figure S4). Meanwhile, a major number of genes showed reverse directed expression changes under IR and the peptides action. Thus, the main effect of peptides at 24 h after ischemia in FC served as a large-scale compensation of gene expression profiles disrupted by ischemia. Furthermore, when we studied the transcriptomic effects of Semax and ACTH(6–9)PGP at 4.5 h after tMCAO, a direct comparison of the data in the ACTH(6–9)PGP group relative to the Semax group identified 315 DEGs in that case [23]. When we found 32 DEGs in a similar comparison at 24 h, we realized that this number was substantially lower than the number observed at 4.5 h after tMCAO. Thus, differences in the action of two peptides in the early hours are more pronounced than a day after ischemia. Interestingly, eight genes (*Egr4*, *Fosb*, *RGD1564664*, *Egr2*, *Igsf9*, *Angptl4*, *FAM187A*, and *Adamts9*) overlapped between the two aforementioned gene sets (Supplementary Figure S5). It can be assumed that these genes not only reflect differences in the effects of ACTH(6–9)PGP and Semax peptides, but are also important as switches in cell metabolism after stroke.

Previously, we found that ACTH(6–9)PGP and Semax peptides induced neuroglial proliferation and vascularization in the rat brain under IR [24]. Here, using RNA-Seq we found a number angiogenesis-, neurogenesis-, and growth factor-related DEGs in FC under peptides action. Many of them were upregulated after administration of Semax or ACTH(6–9)PGP compared to saline under IR conditions (Supplementary Table S12). The results also enable the selection of specific proteins for analysis of their involvement in the mechanisms of peptide regulation. Among them, there may be previously listed Fos, Junb, Jak2, Npas4, Egr2 and other proteins, which may help to validate some key signaling pathways after peptide treatment at the protein level and reveal the potential effects of peptides on ischemic stroke. Also, further functional testing using methods which knockdown genes or inhibit their products will make it possible to understand the post-transcriptional and post-translational level of regulation of gene expression under the action of peptides. It should be noted that all results were obtained using male specimens and, therefore, sex-bias could exist. We plan to overcome all listed limitations in our next studies. In addition, the importance of nanomechanics in the study of ischemic pathological processes, such as strokes, has been shown [62,63]. From a broad perspective, the studies of nanomechanical properties of soft tissues of the brain during IR and the peptides administration can be relevant in the future.

In conclusion, the molecular basis of the action of Semax and ACTH(6–9)PGP peptides includes the compensation of rat brain gene expression profiles disrupted by ischemia, as well as the influence on the genes of the growth, angiogenesis, and neurogenesis systems a day after experimental stroke.

## 5. Conclusions

Our data provide an insight into the activity of related peptides ACTH(6–9)PGP and Semax through the modulation of the transcriptome pattern a day after tMCAO on a genome-wide scale. Individual genes and pathways have been identified as targets of adrenocorticotropic-like peptides. By applying two methods of data comparison, we revealed that the effect of ACTH(6–9)PGP was more similar to Semax than different from it at 24 h after IR. More than 1000 genes were identified as common between the ACTH(6–9)PGP and Semax peptides action. It is possible that overlapped genes form the core of the genetic neuroprotection potential necessary for the recovery processes after a stroke in FC. Among them, genes of the inflammatory and neurotransmitter cluster showed major involvement in the mechanism of action of peptides a day after IR. Moreover, both peptides diminished ischemia-induced changes in the expression of these genes. Specifically, both Semax and ACTH(6–9)PGP activated neurotransmitter and suppressed inflammatory genes, whereas the genetic response initiated by IR activated inflammatory and suppressed neurotransmitter genes. Additionally, neurogenesis-, angiogenesis-, protein kinase- and growth factor-related DEGs were revealed under peptides action. Thus, our results demonstrate the genomic basis of the neuroprotective peptide effects that we previously observed at the histological level in rat brains. It is possible that control of the activity of these genes will provide criteria for the selection of neuroprotective drugs and will shape the prospects for the development of therapy for stroke and its consequences. In the future, further studies are needed at the cellular level to determine the role of these genes at the protein level and to confirm key signaling pathways and functional networks after peptide treatment. Furthermore, comparison with previous data at the 4.5 h post-tMCAO time point showed that the pattern of peptide action on the transcriptome depends on the time elapsed after tMCAO. Thus, our results may be useful for selecting more effective structures for future anti-stroke drugs and appropriate post stroke-time points for their testing.

## Data Availability

Publicly available datasets were analyzed in this study. These data can be found here: [33,34].

## Supplementary Materials

The following are available online at https://www.mdpi.com/article/10.3390/biomedicines12122830/s1: Figure S1. MRI of ischemic foci at 24 h after tMCAO, Figure S2. Real-time reverse transcription polymerase chain reaction (RT-PCR) verification of the RNA-Seq results, Figure S3. Differential gene expression profile at 4.5 h and 24 h after tMCAO in FC under both peptides (Semax, ACTH(6–9)PGP) action, Figure S4. Differential expressed genes that showed enhancing effects of at least one of the peptides (Semax, ACTH(6–9)PGP) on the effect of ischemia itself in the region of frontal cortex (FC) at 24 h after tMCAO, Figure S5. Comparative analysis of ACTH(6–9)PGP action versus Semax action on the transcriptome of the FC at 24 h and 4.5 h after tMCAO, Method S1. Technical information regarding the transient middle cerebral artery occlusion (tMCAO) model procedure, Method S2. Technical information regarding magnetic resonance imaging (MRI) procedure, Table S1. Characterization of the primers used for real-time RT-PCR, Table S2. DEGs in IR24-f vs. SO24-f pairwise comparison, Table S3. DEGs in IS24-f vs. IR24-f pairwise comparison, Table S4. DEGs in IA24-f vs. IR24-f pairwise comparison, Table S5. DEGs that overlapped between IR24-f vs. SO24-f and IS24-f vs. IR24-f pairwise comparisons, Table S6. DEGs that overlapped between IR24-f vs. SO24-f and IA24-f vs. IR24-f pairwise comparisons, Table S7. DEGs that overlapped between IS24-f vs. IR24-f and IA24-f vs. IR24-f pairwise comparisons, Table S8. DEGs that overlapped between IR24-f vs. SO24-f, IS24-f vs. IR24-f and IA24-f vs. IR24-f pairwise comparisons, Table S9. DEGs that overlapped between IS24-f vs. IR24-f and IA24-f vs. IR24-f but not IR24-f vs. SO24-f pairwise comparisons, Table S10. Analysis of pathways associated with DEGs in IR24-f vs. SO24-f, IS24-f vs. IR24-f and IA24-f vs. IR24-f pairwise comparisons at 24 h after tMCAO using DAVID, Table S11. DEGs that associated with inflammatory activity in IS24-f vs. IR24-f or IA24-f vs. IR24-f pairwise comparisons, Table S12. Upregulated DEGs that associated with neurogenesis, angiogenesis, protein kinase, differentiation and growth factors activity in IS24-f vs. IR24-f or IA24-f vs. IR24-f pairwise comparisons, Table S13. DEGs in IA24-f vs. IS24-f and their differential expression in IS24-f vs. IR24-f and IA24-f vs. IR24-f pairwise comparisons.
