# Supplementary material for: ACTH-like Peptides Compensate Rat Brain Gene Expression Profile Disrupted by Ischemia a Day After Experimental Stroke
Source: Biomedicines. 2024 Dec 13;12(12):2830. doi: 10.3390/biomedicines12122830 (PMC11673339; doi:10.3390/biomedicines12122830)
Supplement: Supplementary file 1 [file biomedicines-12-02830-s001.zip › Supplementary Figure S1.pptx]

## Slide 1
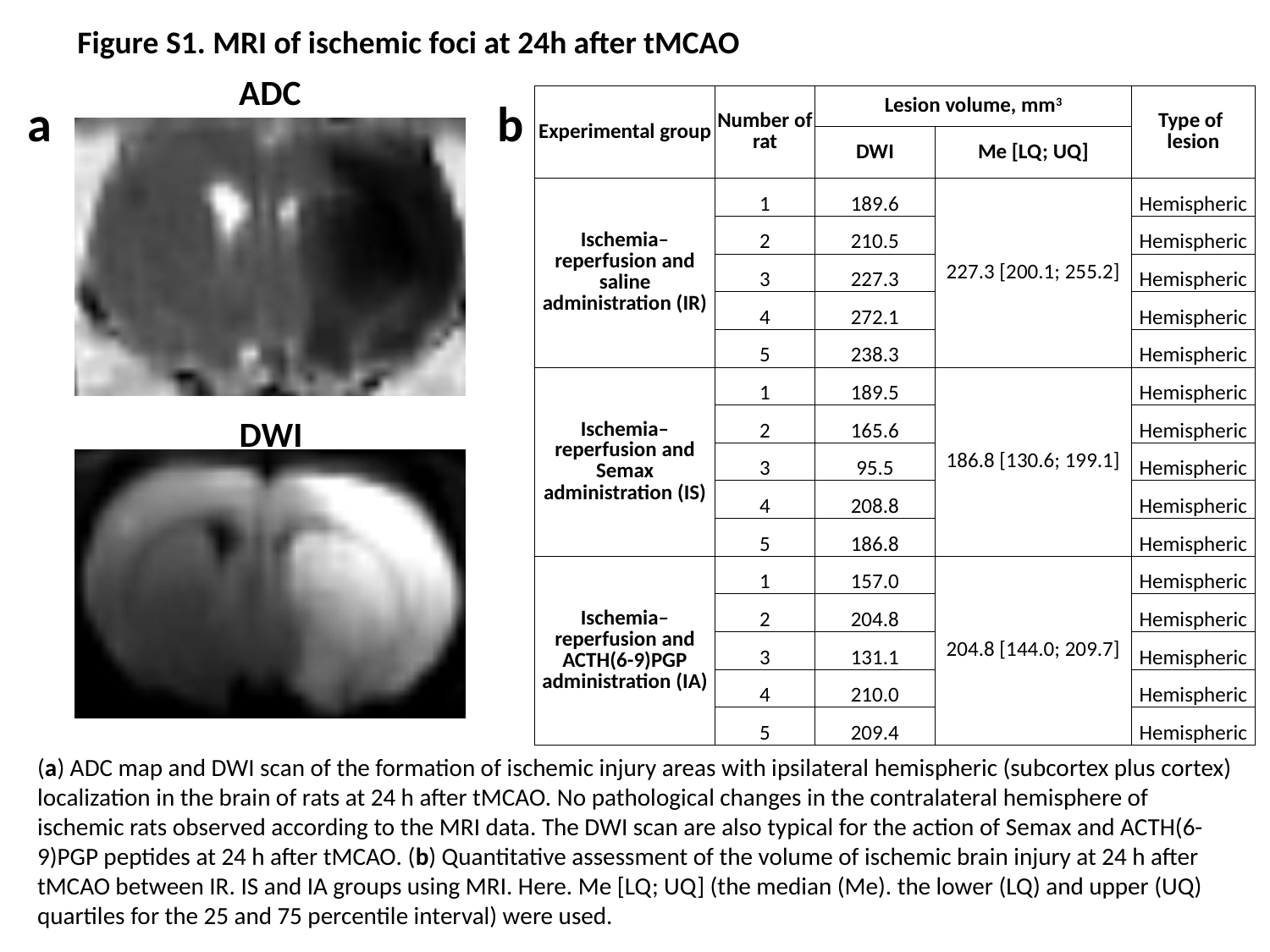

Figure S1. MRI of ischemic foci at 24h after tMCAO
ADC
a
b
| Experimental group | Number of rat | Lesion volume, mm3 | | Type of lesion |
| --- | --- | --- | --- | --- |
| | | DWI | Me [LQ; UQ] | |
| Ischemia–reperfusion and saline administration (IR) | 1 | 189.6 | 227.3 [200.1; 255.2] | Hemispheric |
| | 2 | 210.5 | | Hemispheric |
| | 3 | 227.3 | | Hemispheric |
| | 4 | 272.1 | | Hemispheric |
| | 5 | 238.3 | | Hemispheric |
| Ischemia–reperfusion and Semax administration (IS) | 1 | 189.5 | 186.8 [130.6; 199.1] | Hemispheric |
| | 2 | 165.6 | | Hemispheric |
| | 3 | 95.5 | | Hemispheric |
| | 4 | 208.8 | | Hemispheric |
| | 5 | 186.8 | | Hemispheric |
| Ischemia–reperfusion and ACTH(6-9)PGP administration (IA) | 1 | 157.0 | 204.8 [144.0; 209.7] | Hemispheric |
| | 2 | 204.8 | | Hemispheric |
| | 3 | 131.1 | | Hemispheric |
| | 4 | 210.0 | | Hemispheric |
| | 5 | 209.4 | | Hemispheric |
DWI
(a) ADC map and DWI scan of the formation of ischemic injury areas with ipsilateral hemispheric (subcortex plus cortex) localization in the brain of rats at 24 h after tMCAO. No pathological changes in the contralateral hemisphere of ischemic rats observed according to the MRI data. The DWI scan are also typical for the action of Semax and ACTH(6-9)PGP peptides at 24 h after tMCAO. (b) Quantitative assessment of the volume of ischemic brain injury at 24 h after tMCAO between IR. IS and IA groups using MRI. Here. Me [LQ; UQ] (the median (Me). the lower (LQ) and upper (UQ) quartiles for the 25 and 75 percentile interval) were used.
