# Supplementary material for: ACTH-like Peptides Compensate Rat Brain Gene Expression Profile Disrupted by Ischemia a Day After Experimental Stroke
Source: Biomedicines. 2024 Dec 13;12(12):2830. doi: 10.3390/biomedicines12122830 (PMC11673339; doi:10.3390/biomedicines12122830)
Supplement: Supplementary file 1 [file biomedicines-12-02830-s001.zip › Supplementary Figure S2.pptx]

## Slide 1
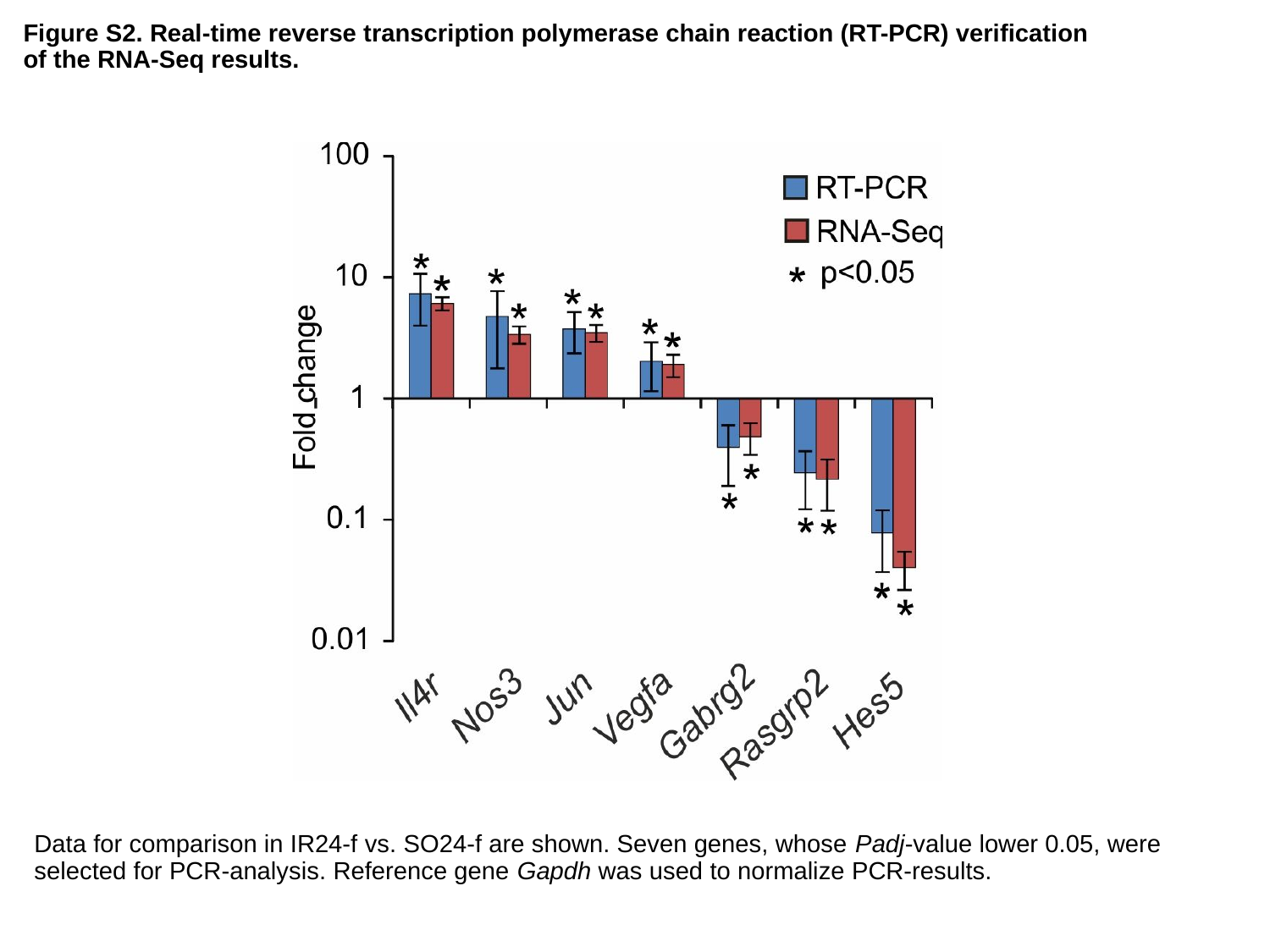

# Figure S2. Real-time reverse transcription polymerase chain reaction (RT-PCR) verification of the RNA-Seq results.
Data for comparison in IR24-f vs. SO24-f are shown. Seven genes, whose Padj-value lower 0.05, were selected for PCR-analysis. Reference gene Gapdh was used to normalize PCR-results.
