# Supplementary material for: ACTH-like Peptides Compensate Rat Brain Gene Expression Profile Disrupted by Ischemia a Day After Experimental Stroke
Source: Biomedicines. 2024 Dec 13;12(12):2830. doi: 10.3390/biomedicines12122830 (PMC11673339; doi:10.3390/biomedicines12122830)
Supplement: Supplementary file 1 [file biomedicines-12-02830-s001.zip › Supplementary Figure S3.pptx]

## Slide 1
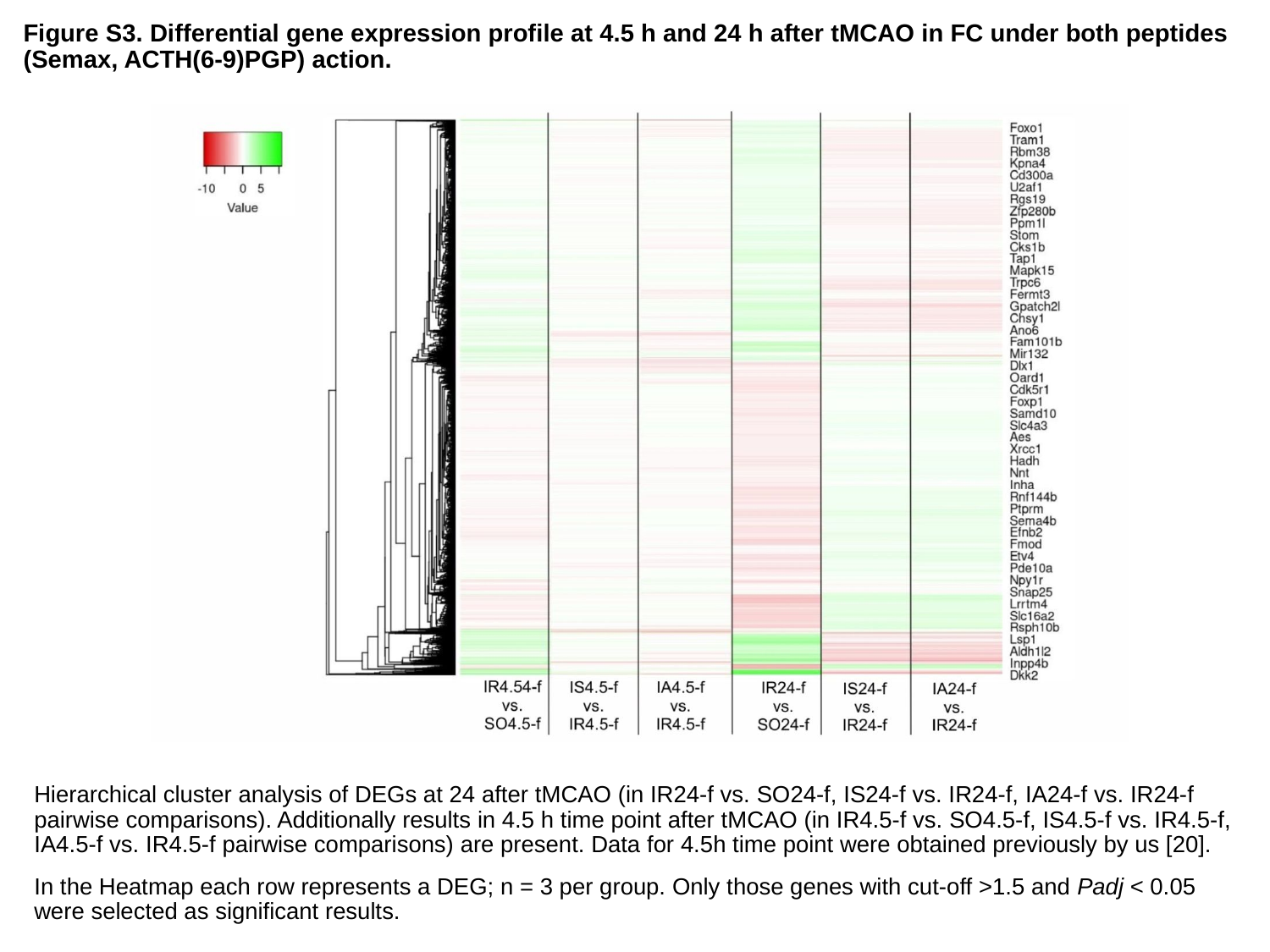

# Figure S3. Differential gene expression profile at 4.5 h and 24 h after tMCAO in FC under both peptides (Semax, ACTH(6-9)PGP) action.
Hierarchical cluster analysis of DEGs at 24 after tMCAO (in IR24-f vs. SO24-f, IS24-f vs. IR24-f, IA24-f vs. IR24-f pairwise comparisons). Additionally results in 4.5 h time point after tMCAO (in IR4.5-f vs. SO4.5-f, IS4.5-f vs. IR4.5-f, IA4.5-f vs. IR4.5-f pairwise comparisons) are present. Data for 4.5h time point were obtained previously by us [20].
In the Heatmap each row represents a DEG; n = 3 per group. Only those genes with cut-off >1.5 and Padj < 0.05 were selected as significant results.
