# Supplementary material for: ACTH-like Peptides Compensate Rat Brain Gene Expression Profile Disrupted by Ischemia a Day After Experimental Stroke
Source: Biomedicines. 2024 Dec 13;12(12):2830. doi: 10.3390/biomedicines12122830 (PMC11673339; doi:10.3390/biomedicines12122830)
Supplement: Supplementary file 1 [file biomedicines-12-02830-s001.zip › Supplementary Figure S4.pptx]

## Slide 1
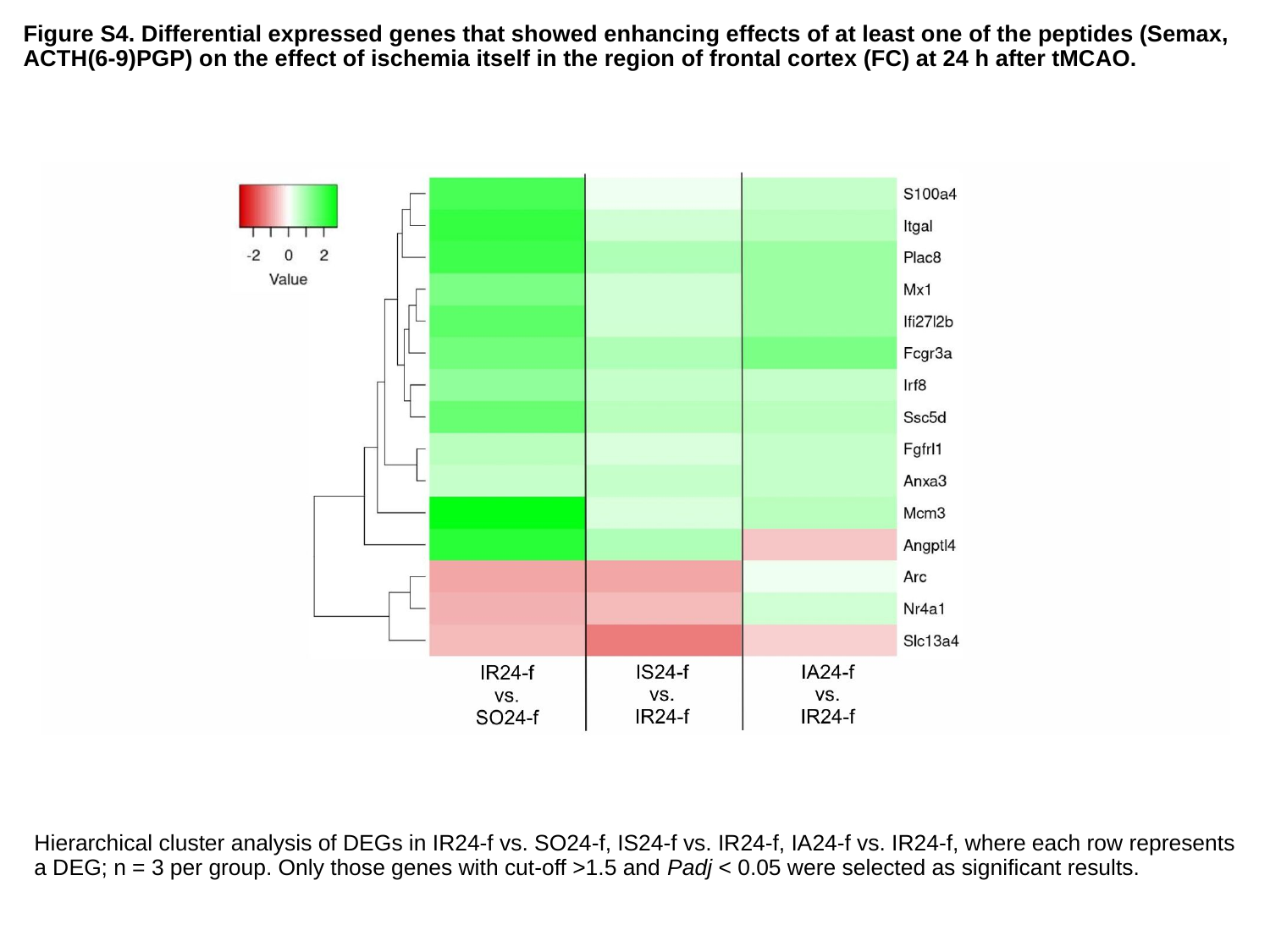

# Figure S4. Differential expressed genes that showed enhancing effects of at least one of the peptides (Semax, ACTH(6-9)PGP) on the effect of ischemia itself in the region of frontal cortex (FC) at 24 h after tMCAO.
Hierarchical cluster analysis of DEGs in IR24-f vs. SO24-f, IS24-f vs. IR24-f, IA24-f vs. IR24-f, where each row represents a DEG; n = 3 per group. Only those genes with cut-off >1.5 and Padj < 0.05 were selected as significant results.
