# Supplementary material for: ACTH-like Peptides Compensate Rat Brain Gene Expression Profile Disrupted by Ischemia a Day After Experimental Stroke
Source: Biomedicines. 2024 Dec 13;12(12):2830. doi: 10.3390/biomedicines12122830 (PMC11673339; doi:10.3390/biomedicines12122830)
Supplement: Supplementary file 1 [file biomedicines-12-02830-s001.zip › Supplementary Figure S5.pptx]

## Slide 1
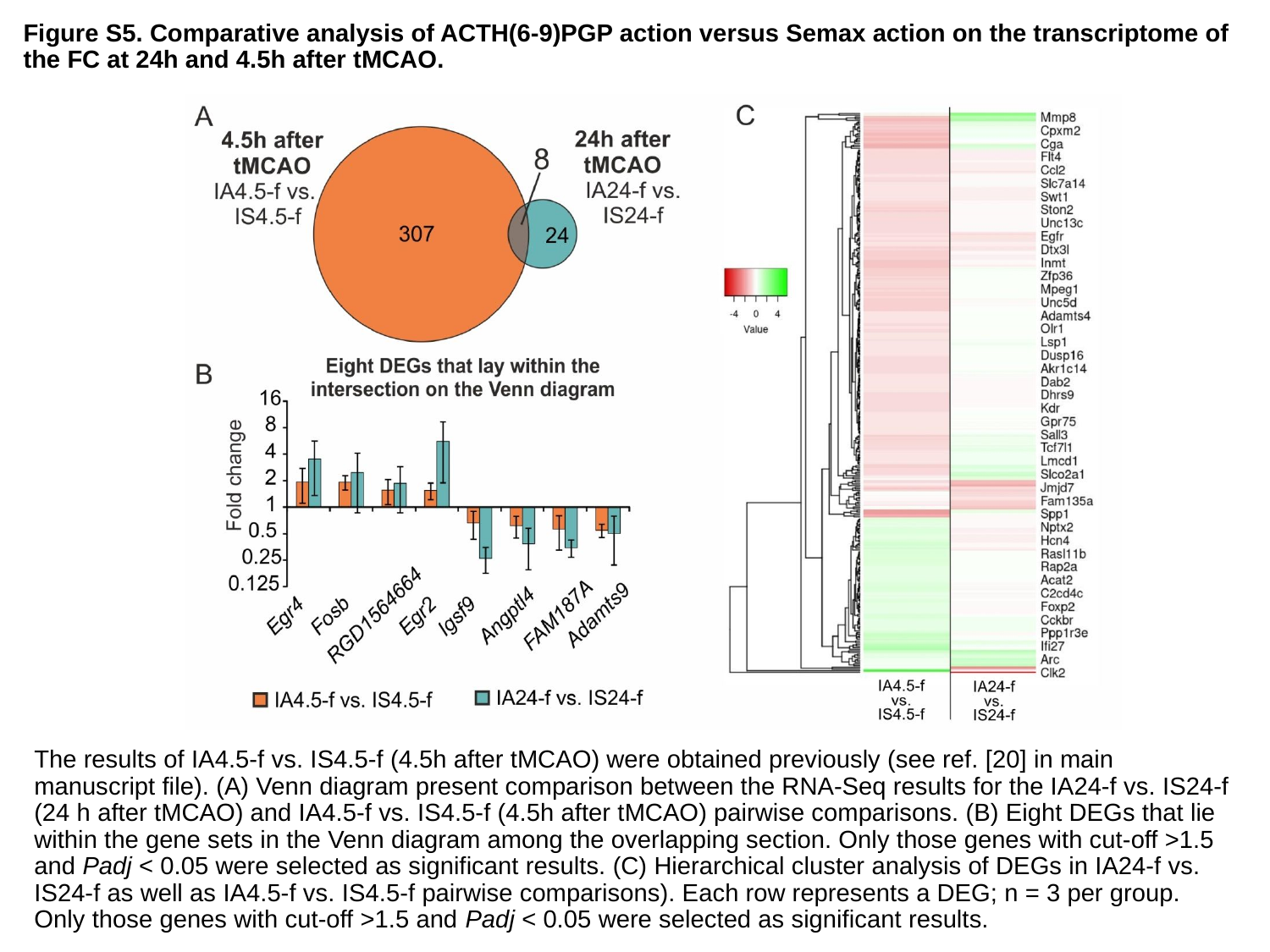

# Figure S5. Comparative analysis of ACTH(6-9)PGP action versus Semax action on the transcriptome of the FC at 24h and 4.5h after tMCAO.
The results of IA4.5-f vs. IS4.5-f (4.5h after tMCAO) were obtained previously (see ref. [20] in main manuscript file). (A) Venn diagram present comparison between the RNA-Seq results for the IA24-f vs. IS24-f (24 h after tMCAO) and IA4.5-f vs. IS4.5-f (4.5h after tMCAO) pairwise comparisons. (B) Eight DEGs that lie within the gene sets in the Venn diagram among the overlapping section. Only those genes with cut-off >1.5 and Padj < 0.05 were selected as significant results. (C) Hierarchical cluster analysis of DEGs in IA24-f vs. IS24-f as well as IA4.5-f vs. IS4.5-f pairwise comparisons). Each row represents a DEG; n = 3 per group. Only those genes with cut-off >1.5 and Padj < 0.05 were selected as significant results.
