# Supplementary material for: ACTH-like Peptides Compensate Rat Brain Gene Expression Profile Disrupted by Ischemia a Day After Experimental Stroke
Source: Biomedicines. 2024 Dec 13;12(12):2830. doi: 10.3390/biomedicines12122830 (PMC11673339; doi:10.3390/biomedicines12122830)
Supplement: Supplementary file 1 [file biomedicines-12-02830-s001.zip › Supplementary Method S1.pptx]

## Slide 1
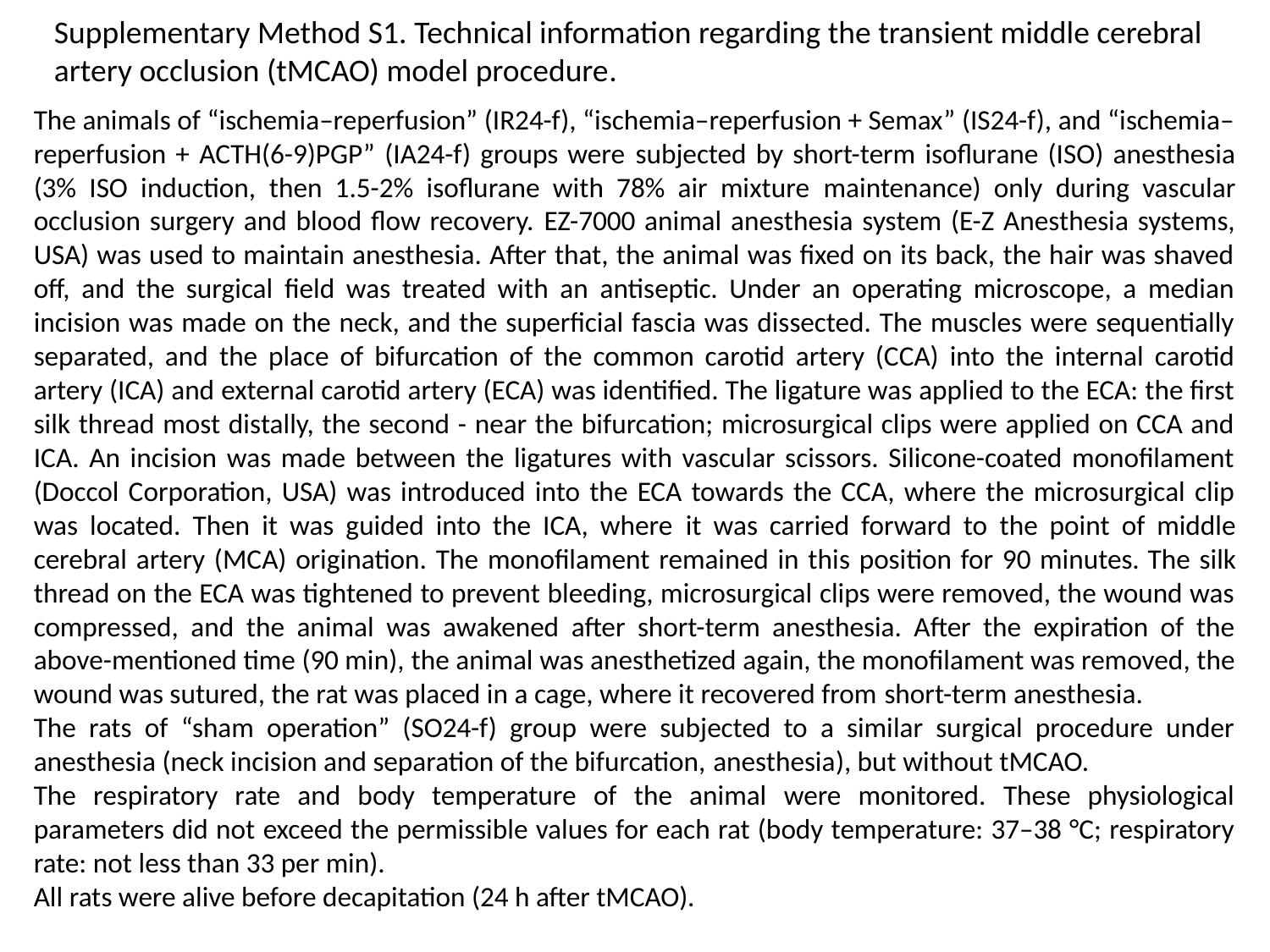

Supplementary Method S1. Technical information regarding the transient middle cerebral artery occlusion (tMCAO) model procedure.
The animals of “ischemia–reperfusion” (IR24-f), “ischemia–reperfusion + Semax” (IS24-f), and “ischemia–reperfusion + ACTH(6-9)PGP” (IA24-f) groups were subjected by short-term isoflurane (ISO) anesthesia (3% ISO induction, then 1.5-2% isoflurane with 78% air mixture maintenance) only during vascular occlusion surgery and blood flow recovery. EZ-7000 animal anesthesia system (E-Z Anesthesia systems, USA) was used to maintain anesthesia. After that, the animal was fixed on its back, the hair was shaved off, and the surgical field was treated with an antiseptic. Under an operating microscope, a median incision was made on the neck, and the superficial fascia was dissected. The muscles were sequentially separated, and the place of bifurcation of the common carotid artery (CCA) into the internal carotid artery (ICA) and external carotid artery (ECA) was identified. The ligature was applied to the ECA: the first silk thread most distally, the second - near the bifurcation; microsurgical clips were applied on CCA and ICA. An incision was made between the ligatures with vascular scissors. Silicone-coated monofilament (Doccol Corporation, USA) was introduced into the ECA towards the CCA, where the microsurgical clip was located. Then it was guided into the ICA, where it was carried forward to the point of middle cerebral artery (MCA) origination. The monofilament remained in this position for 90 minutes. The silk thread on the ECA was tightened to prevent bleeding, microsurgical clips were removed, the wound was compressed, and the animal was awakened after short-term anesthesia. After the expiration of the above-mentioned time (90 min), the animal was anesthetized again, the monofilament was removed, the wound was sutured, the rat was placed in a cage, where it recovered from short-term anesthesia.
The rats of “sham operation” (SO24-f) group were subjected to a similar surgical procedure under anesthesia (neck incision and separation of the bifurcation, anesthesia), but without tMCAO.
The respiratory rate and body temperature of the animal were monitored. These physiological parameters did not exceed the permissible values for each rat (body temperature: 37–38 °C; respiratory rate: not less than 33 per min).
All rats were alive before decapitation (24 h after tMCAO).
