# Supplementary material for: ACTH-like Peptides Compensate Rat Brain Gene Expression Profile Disrupted by Ischemia a Day After Experimental Stroke
Source: Biomedicines. 2024 Dec 13;12(12):2830. doi: 10.3390/biomedicines12122830 (PMC11673339; doi:10.3390/biomedicines12122830)
Supplement: Supplementary file 1 [file biomedicines-12-02830-s001.zip › Supplementary Method S2.pptx]

## Slide 1
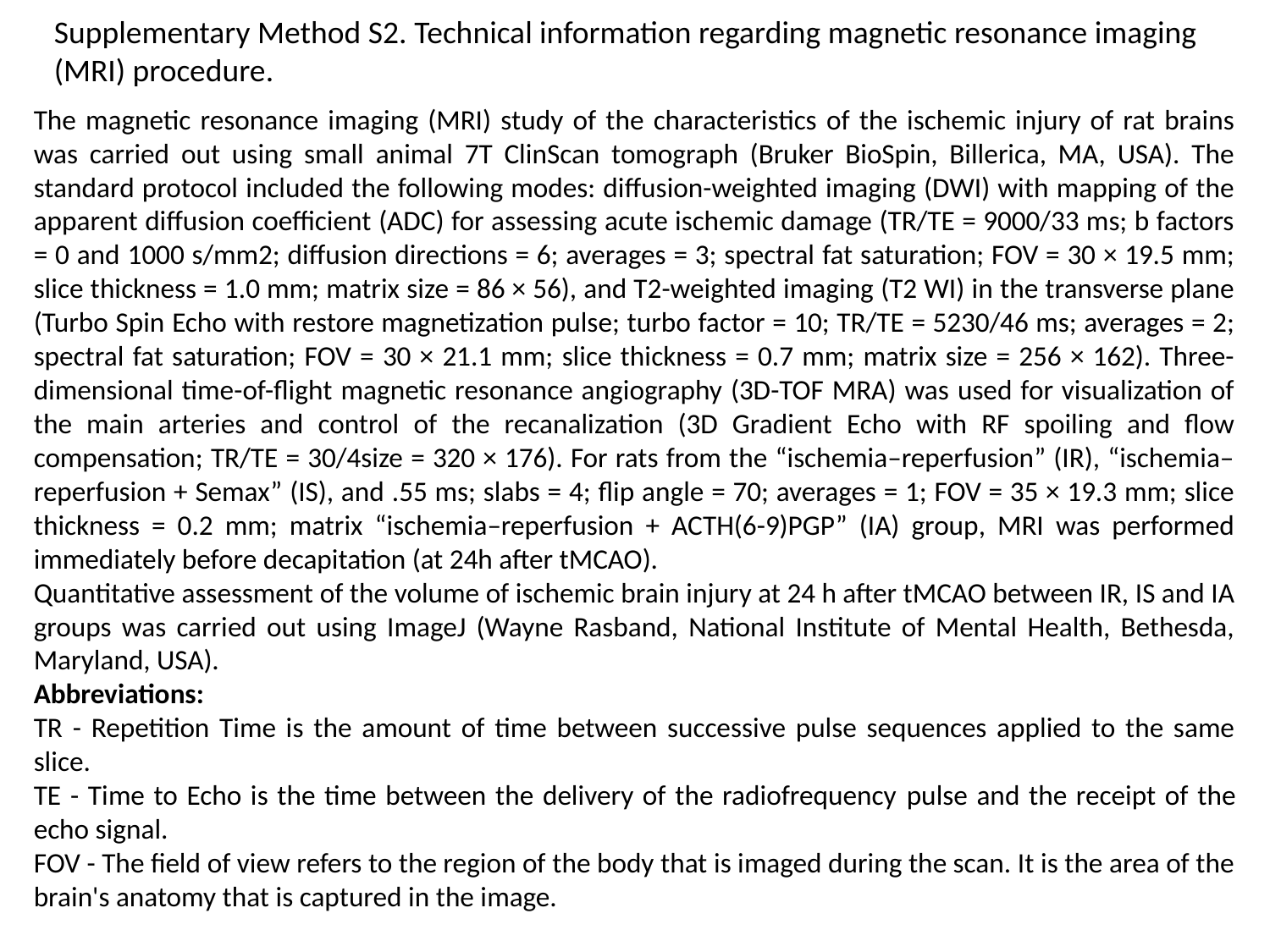

Supplementary Method S2. Technical information regarding magnetic resonance imaging (MRI) procedure.
The magnetic resonance imaging (MRI) study of the characteristics of the ischemic injury of rat brains was carried out using small animal 7T ClinScan tomograph (Bruker BioSpin, Billerica, MA, USA). The standard protocol included the following modes: diffusion-weighted imaging (DWI) with mapping of the apparent diffusion coefficient (ADC) for assessing acute ischemic damage (TR/TE = 9000/33 ms; b factors = 0 and 1000 s/mm2; diffusion directions = 6; averages = 3; spectral fat saturation; FOV = 30 × 19.5 mm; slice thickness = 1.0 mm; matrix size = 86 × 56), and T2-weighted imaging (T2 WI) in the transverse plane (Turbo Spin Echo with restore magnetization pulse; turbo factor = 10; TR/TE = 5230/46 ms; averages = 2; spectral fat saturation; FOV = 30 × 21.1 mm; slice thickness = 0.7 mm; matrix size = 256 × 162). Three-dimensional time-of-flight magnetic resonance angiography (3D-TOF MRA) was used for visualization of the main arteries and control of the recanalization (3D Gradient Echo with RF spoiling and flow compensation; TR/TE = 30/4size = 320 × 176). For rats from the “ischemia–reperfusion” (IR), “ischemia–reperfusion + Semax” (IS), and .55 ms; slabs = 4; flip angle = 70; averages = 1; FOV = 35 × 19.3 mm; slice thickness = 0.2 mm; matrix “ischemia–reperfusion + ACTH(6-9)PGP” (IA) group, MRI was performed immediately before decapitation (at 24h after tMCAO).
Quantitative assessment of the volume of ischemic brain injury at 24 h after tMCAO between IR, IS and IA groups was carried out using ImageJ (Wayne Rasband, National Institute of Mental Health, Bethesda, Maryland, USA).
Abbreviations:
TR - Repetition Time is the amount of time between successive pulse sequences applied to the same slice.
TE - Time to Echo is the time between the delivery of the radiofrequency pulse and the receipt of the echo signal.
FOV - The field of view refers to the region of the body that is imaged during the scan. It is the area of the brain's anatomy that is captured in the image.
